# Compounds from *Terminalia mantaly* L. (Combretaceae) Stem Bark Exhibit Potent Inhibition against Some Pathogenic Yeasts and Enzymes of Metabolic Significance

**DOI:** 10.3390/medicines4010006

**Published:** 2017-01-24

**Authors:** Marthe Aimée Tchuente Tchuenmogne, Thierry Ngouana Kammalac, Sebastian Gohlke, Rufin Marie Toghueo Kouipou, Abdulselam Aslan, Muslum Kuzu, Veysel Comakli, Ramazan Demirdag, Silvère Augustin Ngouela, Etienne Tsamo, Norbert Sewald, Bruno Ndjakou Lenta, Fabrice Fekam Boyom

**Affiliations:** 1 Laboratory of Natural Products and Organic Synthesis, Department of Organic Chemistry, Faculty of Science, University of Yaoundé 1, P.O. Box 812, Yaoundé, Cameroon; tch_aimee@yahoo.fr (M.A.T.T.); sngouela@yahoo.fr (S.A.N.); tsamoet@yahoo.fr (E.T.); 2 Antimicrobial & Biocontrol Agents Unit, Laboratory for Phytobiochemistry and Medicinal Plants Studies, Department of Biochemistry, Faculty of Science, University of Yaoundé I, P.O. Box 812, Yaoundé, Cameroon; ngouanathi@yahoo.com (T.N.K.); toghueo.rufin@yahoo.fr (R.M.T.K.); 3 Chemistry Department, Organic and Bioorganic Chemistry, Bielefeld University, P.O. Box 100131, D-33501 Bielefeld, Germany; sebastian.gohlke@uni-bielefeld.de (S.G.); norbert.sewald@uni-bielefeld.de (N.S.); 4 Faculty of Engineering, Department of Industrial Engineering, Giresun University, 28200 Giresun, Turkey; abdulselam@hotmail.de; 5 Faculty of Pharmacy, Department of Basic Pharmaceutical Sciences, Agrı Ibrahim Cecen University, 04100 Agri, Turkey; mkuzu@agri.edu.tr; 6 School of Health, Department of Nutrition and Dietetics, Agrı Ibrahim Cecen University, 04100 Agri, Turkey; veysel_comakli@hotmail.com (V.C.); r.demirdag@hotmail.com (R.D.); 7 Department of Chemistry, Higher Teacher Training College, University of Yaoundé 1, Yaoundé, Cameroon

**Keywords:** *Terminalia mantaly*, Combretaceae, anti-yeast, enzyme inhibitors

## Abstract

**Background**: Pathogenic yeasts resistance to current drugs emphasizes the need for new, safe, and cost-effective drugs. Also, new inhibitors are needed to control the effects of enzymes that are implicated in metabolic dysfunctions such as cancer, obesity, and epilepsy. **Methods:** The anti-yeast extract from *Terminalia mantaly* (Combretaceae) was fractionated and the structures of the isolated compounds established by means of spectroscopic analysis and comparison with literature data. Activity was assessed against *Candida albicans*, *C. parapsilosis* and *C. krusei* using the microdilution method, and against four enzymes of metabolic significance: glucose-6-phosphate dehydrogenase, human erythrocyte carbonic anhydrase I and II, and glutathione *S*-transferase. **Results:** Seven compounds, 3,3′-di-*O*-methylellagic acid 4′-*O*-α-rhamnopyranoside; 3-*O*-methylellagic acid; arjungenin or 2,3,19,23-tetrahydroxyolean-12-en-28-oïc acid; arjunglucoside or 2,3,19,23-tetrahydroxyolean-12-en-28-oïc acid glucopyranoside; 2α,3α,24-trihydroxyolean-11,13(18)-dien-28-oïc acid; stigmasterol; and stigmasterol 3-*O*-β-d-glucopyranoside were isolated from the extract. Among those, 3,3′-di-*O*-methylellagic acid 4′-*O*-α-rhamnopyranoside, 3-*O*-methylellagic acid, and arjunglucoside showed anti-yeast activity comparable to that of reference fluconazole with minimal inhibitory concentrations (MIC) below 32 µg/mL. Besides, Arjunglucoside potently inhibited the tested enzymes with 50% inhibitory concentrations (IC_50_) below 4 µM and inhibitory constant (Ki) <3 µM. **Conclusions:** The results achieved indicate that further SAR studies will likely identify potent hit derivatives that should subsequently enter the drug development pipeline.

## 1. Introduction

Fungal diseases affect 3–4 million people worldwide every year. Of particular importance, the increasing resistance of pathogenic opportunistic yeasts to current drugs is a serious concern and has attracted the attention of the scientific community. New, safe, and cost-effective drugs of natural or synthetic origin are therefore actively being researched [1]. Recent epidemiological data highlight the increasing burden of pathogenic yeasts on people in poor settings [2,3,4]. *Candida* species and *Cryptococcus neoformans* are the major pathogenic yeasts and only a few antifungal drugs have been developed so far to treat the invasive infections they cause [5,6]. Medicinal plants have shown credibility as sources of treatment for infectious diseases [7]. In Cameroon, extracts from medicinal plants such as *Terminalia mantaly* (Combretaceae) are widely used by traditional healers to control diverse infections or associated symptoms, including but not limited to dysentery, gastroenteritis, hypertension, diabetes, and oral, dental, cutaneous and genital affections [8]. Previous studies on the extracts of this plant have showed antibacterial and antifungal properties, but their chemical compositions have not yet been determined [9]. However, phytochemical studies of other species of the genus *Terminalia* have reported the presence of flavonoids, terpenoids and their glycosides derivatives, tannins, flavonones and chalcones [10,11,12,13,14,15,16,17,18]. In spite of the work done on *Terminalia* species, no investigation has been attempted yet on the enzyme inhibition properties of their extracts and constituents targeting glucose-6-phosphate dehydrogenase, carbonic anhydrase and glutathione *S*-transferase.

Glucose-6-phosphate dehydrogenase (G6PD; EC 1.1.1.49) is an enzyme that catalyzes the reaction of glucose-6-phosphate into phosphogluconate, which is the rate-limiting first step of the pentose phosphate pathway. The end products of this pathway are ribose-5-phosphate and NADPH. Ribose-5-phosphate is used in DNA or RNA synthesis in cell reproduction, and NADPH is used as a coenzyme for the enzymes participating in the production of reduced glutathione. Given its role in cell growth, this enzyme is of high importance to mammal cells [19,20]. However, several studies have shown that this enzyme has an important role in the pathology of some diseases like cancer, hypertension, heart failure and type 2 diabetes. G6PD activity increases in cancer cells and its inhibition results in decreased cell proliferation and induction of apoptosis. For example, 6-aminonicotinamide, which is an inhibitor of G6PD, has found use in the therapy of various tumors in the past [21].

The carbonic anhydrase (CA; carbonate hydro-lyase, EC 4.2.1.1) enzyme exists commonly in living organisms, and has various isoenzymes according to conditions and necessities of the medium. It is one of the most studied enzymes and CAI and CAII are the most common isoenzymes [22]. In many physiological and pathological processes, CAs catalyze the conversion of CO_2_ to HCO_3_^−^ and H^+^. In addition, CA inhibitors may be used in the treatment of various diseases such as oedema, glaucoma, obesity, cancer, epilepsy and osteoporosis [23].

In living cells, the deleterious effects of free radicals and their intermediates are eliminated or minimized by various enzymatic and non-enzymatic defense systems. Enzymatic defense is provided by several enzymes such as glutathione *S*-transferase (GST), glutathione reductase, glutathione peroxidase, superoxide dismutase, and catalase [24]. The GSTs (EC 2.5.1.18) are a group of multifunctional enzymes that play an important role in animal metabolism [25]. GSTs are important for the fight against cancer because of their interactions with carcinogens and chemotherapeutic agents. They are the target of antiasthmatic and antitumor drugs [26]. Production of excessive amounts of GST in mammalian tumor cells leads to resistance to some anticancer drugs and chemical carcinogens [27].

The reduction of drug effects in tumor cells is an important factor limiting the therapeutic efficacy of an antineoplastic agent. Over time, the development of this resistance is associated with glutathione (GSH) and glutathione *S*-transferase (GST) levels in cells and changes in permeability to the drug. In this regard, G6PD, CA I, II or GST inhibitors may be useful because of their several applications, in particular for the treatment of glaucoma, epilepsy, cancer and as diuretics.

In our search for bioactive secondary metabolites from Cameroonian medicinal plants, we have investigated the MeOH extract of the stem bark of *T. mantaly* L. (Combretaceae) that previously showed anti-yeast activity. We report in this paper the inhibitory potential of compounds isolated from this extract against some pathogenic yeasts and some enzymes of metabolic significance.

## 2. Materials and Methods

### 2.1. General Experimental Procedures

The physicochemical analyses of the isolated natural products were essentially performed as previously described [28]. Optical rotations were measured on a JASCO digital polarimeter (model DIP-3600, JASCO, Tokyo, Japan). UV spectra were determined on a Spectronic Unicam spectrophotometer (Thermo Scientific, Waltham, MA, USA). IR spectra were determined on a JASCO Fourier transform IR-420 spectrometer (JASCO, Tokyo, Japan). ^1^H and ^13^C NMR spectra were run on a Bruker spectrometer (Bruker Corporation, Brussels, Belgium) equipped with 5 mm ^1^H and ^13^C probes operating at 500 and 125 MHz, respectively, with Tetramethylsilane (TMS) as internal standard. Silica gel 230–400 mesh (Merck, Bielefeld, Germany) and silica gel 70–230 mesh (Merck) were used for flash and column chromatography while precoated aluminum-backed silica gel 60 F254 sheets were used for TLC. Spots were visualized under UV light (254 and 365 nm) or using MeOH–H_2_SO_4_ reagent.

### 2.2. Plant Material

The stem bark of *T. mantaly* (Combretaceae) was collected in Yaoundé, Cameroon in May 2012 and identified at the Cameroon National Herbarium where a voucher specimen is deposited under the reference N° 64212/HNC (*T. mantaly* H. Perrier).

### 2.3. Microbial Isolates

Yeast isolates were kindly provided by the Laboratory of Clinical Biology, Yaoundé Central Hospital and consisted of clinical isolates of *C. albicans*, *C. krusei* and *C. parapsilosis.* Yeasts were maintained at room temperature and cultured at 37 °C for 24 h on Sabouraud Dextrose Agar (Oxoid, Drongen, Belgium) slants prior to use.

### 2.4. Plant Extraction and Screening of Anti-Yeast Activity

The harvested *T. mantaly* stem bark was dried at room temperature and ground using a blender. The powdered stem bark (7 kg) was extracted at r.t. with MeOH (48 h). The extract was concentrated under vacuum to afford a dark residue (250 g). Minimal inhibitory concentration (MIC) of the extract was determined according to the CLSI M27-A3 [6] protocol with little modifications. The RPMI 1640 supplemented with 2% glucose was used as culture medium. Briefly for the fungal susceptibility tests, 50 µL of serially two-fold diluted concentrations of the crude extract were added in triplicate wells of a 96-wells microtiter plate. Fifty µL of fungal inocula standardized to a final concentration of 0.5–2.5 × 10^3^ CFU/mL were then individually added in each well of the plate. Plant crude extract and the positive control (fluconazole) at concentrations of 0.12 to 64 µg/mL were tested in a final volume of 100 µL. So-prepared plates were incubated at 37 °C for 48 h. MIC value was subsequently determined through macroscopic observation of plate wells, and was defined as the lowest concentration of the inhibitor that allowed no visible growth of the microorganism after overnight incubation compared to the growth control.

### 2.5. Isolation of Compounds and Screening for Activity

A portion of 180 g of the extract was subjected to medium pressure flash chromatography over silica gel (Merck, 70–230 mesh) using mixtures *n*-hexane-EtOAc of increasing polarity ((70:30)–(0:100)) and EtOAc–MeOH ((95:5)–(50:50)), resulting in the collection of 75 fractions of 500 mL each, which were combined on the basis of TLC analysis to create four fractions labeled T_1_–T_4_. Fraction T_1_ (*m* = 14.4 g) obtained from the mixtures of *n*-hexane-EtOAc (100:0 to 70:30) was subjected to silica gel column chromatography, eluted with *n*-hexane-EtOAc, and yielded oils stigmasterol (23 mg) and arjungenin (7 mg). From fraction T_2_ (*m* = 60.3 g), eluted with *n*-hexane-EtOAc ((50:50)–(25:75)), stigmasterol 3-*O*-β-d-glucopyranoside (12 mg), arjungenin (17 mg), 2α,3α,24-trihydroxyolean-11,13(18)-dien-28-oic acid (5.0 mg) and arjunglucoside (6 mg) were isolated. Column chromatography of fraction T_3_ (*m* = 55.0 g) on silica gel and eluted with the mixtures of EtOAc–MeOH ((100:0)–(85:15)), yielded 3,3′-di-*O*-methylellagic acid 4′-*O*-α-rhamnopyranoside (32 mg), arjungenin (12.0 mg), arjunglucoside (3.5 mg), 2α,3α,24-trihydroxyolean-11,13(18)-dien-28-oic acid (3.5 mg) and a dark mixture that was subjected to column chromatography on Sephadex LH-20 with MeOH as an isocratic eluent and yielded 3-*O*-methyl ellagic acid (12.5 mg). Fraction T_4_ (*m* = 56.18 g) obtained with the solvent system of EtOAc–MeOH (85:15 to 65:35) was a complex mixture and thus was not studied. All the isolated compounds were screened as described above for anti-yeast activity, and as described below for enzyme inhibition activities.

### 2.6. Purification of Glucose 6-Phosphate Dehydrogenase and Activity Determination

G6PD was purified from the gill tissue of Lake Van fish according to Kuzu et al. [29], and the enzyme activity was determined spectrophotometrically using a Shimadzu UV-1800 spectrophotometer (Shimadzu, Tokyo, Japan) at 25 °C, according to the method described by Beutler [30] and based on the principle of the reduction of NADP^+^ to NADPH in the presence of glucose 6-phosphate and absorbance recorded at 340 nm.

### 2.7. Purification of Carbonic Anhydrase Isoenzymes by Affinity Chromatography and Activity Determination

The purification of hCA I and II isozymes was performed with a simple one step method by a Sepharose-4B anilinesulphanilamide affinity column chromatograph as previously described [31]. Briefly, CNBr activated Sepharose-4B was washed with ddH_2_O and tyrosine further attached to the activated gel as a spacer arm and finally diazotized sulphanilamide clamped with tyrosine molecule as ligand. The homogenate was applied to the prepared Sepharose-4B-l-Tyrosine Sulphanilamide affinity column equilibrated with 25 mM Tris–HCl/0.1 M Na_2_SO4 (pH 8.7) (Sigma-Aldrich, Taufkirchen, Germany). The affinity gel was washed with 25 mM Tris–HCl/22 mM Na_2_SO4 (pH 8.7).

The esterase activity was assessed following the change in absorbance of 4-nitrophenylacetate (NPA) to 4-nitrophenylate ion at 348 nm over a period of 3 min at 25 °C using a Beckman Coulter UV-VIS spectrophotometer (Beckman Coulter, Atlanta, GA, USA) according to the method described by Verpoorte et al. [32].

### 2.8. Purification of Glutathione S-Transferase Enzyme and Activity Determination

Firstly, heamolysate from human erythrocytes was prepared according to the method of Hunaiti et al. [33]. The prepared heamolysate was directly applied to the glutathione-agarose affinity column and washed with 10 mM KH_2_PO_4_ and 0.1 M KCl (pH 8.0) (Sigma-Aldrich). The washing procedure was monitored on a spectrophotometer through equal–to–blind absorbance values. After the column was stabilized, the enzyme was purified by gradient elution at +4 °C [24,34]. Elution solvent was prepared from a solvent gradient containing 50 mM Tris–HCl and (1.25–10 mM GSH, pH 9.5). Thereafter, 1-chloro-2,4-dinitrobenzene (Sigma-Aldrich) was used to determine GST enzyme activity. In fact the complex obtained using dinitrobenzene *S*-glutathione (DNB-SG) displays maximum absorbance at 340 nm. Activity measurements were thus carried out using the absorbance increment at this wavelength. [35].

### 2.9. In Vitro Inhibition and Kinetic Studies

To determine the effects of compounds on enzymes, enzyme activities were measured with saturated substrate concentration and five different inhibitor concentrations. The 50% inhibitory concentrations (IC_50_) were determined by plotting curves of percent inhibition versus compound concentration. Results are reported as IC_50_ values. Ki constants were calculated using the Cheng-Prusoff equation [36].

## 3. Results and Discussion

The methanol extract of the stem bark of *T. mantaly* was screened for anti-yeast activity in vitro against three clinical isolates consisting of *C. albicans*, *C. krusei* and *C. parapsilosis.* The crude extract exhibited good activity with MIC values of 24 µg/mL against *C. parapsilosis* and 39 µg/mL against *C. albicans* and *C. krusei* (Table 1).

**Figure 1:**
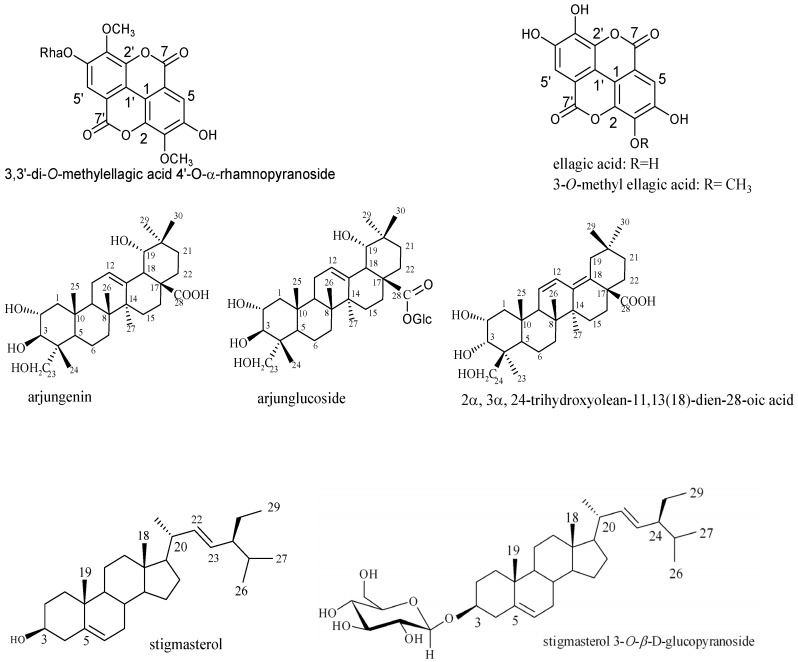
Structures of the isolated compounds from *Terminalia mantaly* (Combretaceae). The isolated compounds were tested against pathogenic yeast isolates and enzymes of metabolic significance. 3,3′-di-*O*-methylellagic acid 4′-*O*-α-rhamnopyranoside: IC_50_ = 39 µg/mL *C. parapsilosis*; 9.7 µg/mL *C. albicans*; >5000 µg/mL *C. krusei*; CAI: IC_50_ = 53.31 µM, Ki = 44.11 µM; CAII: IC_50_ = 69.11 µM, Ki = 55.78 µM; GST: IC_50_ = 63.01 µM, Ki = 42.00 µM. 3-*O*-methyl ellagic acid: IC_50_ = 78 µg/mL *C. parapsilosis*; 156 µg/mL *C. albicans*; 19.5 µg/mL *C. krusei*. arjungenin: *C. parapsilosis*, *C. albicans*, *krusei*: IC_50_ > 5000 µg/mL; CAI: IC_50_ = 86.64 µM, Ki = 71.68 µM; GST: IC_50_ = 1.51 µM, Ki = 1.00 µM; arjunglucoside: IC_50_ = 39 µg/mL *C. parapsilosis*; 9.7 µg/mL *C. albicans*; 312 µg/mL *C. krusei*; G6PD: IC_50_ = 1.84 µM, Ki = 0.19 µM; CAI: IC_50_ = 3.28 µM, Ki = 2.72 µM; CAII: IC_50_ = 1.28 µM, Ki = 1.03 µM; GST: IC_50_ = 1.84 µM, Ki = 1.23 µM. 2α,3α,24-trihydroxyolean-11,13(18)-dien-28-oic acid: IC_50_ > 5000 µg/mL *C. parapsilosis*, *C. albicans*, *C. krusei*.

The flash chromatography of the crude extract generated four fractions exhibiting varying antifungal activities. As shown in Table 1, fraction T3 was the most active, with activity magnification over 1950 times against *C. krusei* (MIC = 0.02 µg/mL), 150 times against *C. parapsilosis* (MIC = 0.16 µg/mL), and over 60 times against *C. albicans* (0.64 µg/mL), compared to the crude extract (MIC = 24–39 µg/mL)*. C. krusei* was the most susceptible isolate to fraction T3. Compounds 3,3′-di-*O*-methylellagic acid-4′-*O*-α-rhamnopyranoside, 3-*O*-methylellagic acid, arjungenin, arjunglucoside, and 2α,3α,24-trihydroxyolean-11,13(18)-dien-28-oic acid that were all found in fraction T3 were also tested for biological activity (Table 1; Figure 1). Overall, they showed drastically reduced potency against the tested yeasts compared to the mother fraction T3, indicating that fractionation has negatively affected the biological activity. Compounds stigmasterol and stigmasterol 3-*O*-β-d-glucopyranoside were not tested due to reduced solubility in the culture medium. Among the tested compounds, 3,3′-di-*O*-methylellagic acid-4′-*O*-α-rhamnopyranoside and arjunglucoside showed the best potency against *C. albicans* with an MIC of 9.7 µg/mL. They also moderately inhibited *C. parapsilosis* with an MIC of 39 µg/mL. In addition, compound 3-*O*-methylellagic acid inhibited *C. krusei* with an MIC of 19.5 µg/mL.

## 4. NMR Spectral Data of the Tested Compounds

The physicochemical profiles of the isolated compounds were acquired following previously described approaches [37,38,39,40].

*3,3*′*-di-O-methylellagic acid 4*′*-O-*α*-rhamnopyranoside*. Yellowish powder; molecular formula C_21_H_18_O_12_; ESI-MS: [M + Na]^+^ *m*/*z* 485,049 ^1^H-NMR (300 MHz, DMSO-*d*_6_): δ_H_ 1.13 (3 H, d, CH_3_, H-6′′), 3.54 (1 H, q, *J* = 8.0 and 12.0 Hz, H-5′′), 4.01 (1 H, t, H-4′′), 4.04 (3 H, s, OMe-3), 4.72 (1 H, brd, *J* = 8.0 Hz, H-3′′), 4.94 (1 H, brd, *J* = 4.0 Hz, H-2′′), 5.47 (1 H, brs, H-1′′), 7.52 (1 H, s, H-5), 7.73 (1 H, s, H-5′); ^13^C-NMR (125 MHz, DMSO-*d*_6_), aglycone moiety: δ_C_ 113.4 (C-1), 140.5 (C-2), 141.8 (C-3), 153.1 (C-4), 111.9 (C-5), 113.4 (C-6), 159.1 (C-7), 114.7 (C-1′), 136.6 (C-2′), 142.2 (C-3′), 146.9 (C-4′), 112.0 (C-5′), 107.4 (C-6′), 159.1 (C-7′); rhamnose moiety: 100.5 (C-1′′), 70.4 (C-2′′), 70.5 (C-3′′), 72.2 (C-4′′), 70.3 (C-5′′), 18.3 (C-6′′) and 61.4 (C-3, OMe).

*3-O-methyl ellagic acid*. Yellowish powder; molecular formula C_15_H_8_O_8_; ESI-MS: [M − H]^−^ *m*/*z* 315, ^1^H-NMR (DMSO-*d*_6_): *δ* 7.50 (1 H, s, H-5), 7.44 (1 H, s, H-5′), 4.02 (3 H, s, 3-OMe). ^13^C-NMR (DMSO-*d*_6_): *δ* 158.9 (C-7), 158.6 (C-7′), 152.2 (C-4), 148.2 (C-4′), 141.7 (C-2), 140.0 (C-3), 139.8 (C-3′), 136.1 (C-2′), 112.4(C-1′), 112.1 (C-6), 111.7 (C-1), 111.3 (C-5), 110.1 (C-5′), 107.2 (C-6′), 60.8 (3-OMe).

*Arjungenin or 2,3,19,23-tetrahydroxyolean-12-en-28-oic acid*. White powder; molecular formula C_30_H_48_O_6_; ESI-MS: [M + Na]^+^ *m*/*z* 527,322. ^1^H-NMR (300 MHz, DMSO-*d*_6_): δ_H_ 1.23, 1.09, 0.90, 0.88, 0.84 and 0.65 (each 3 H, s); 2.92 (1 H, brs, H-18); 2.86 (1 H, d, *J* = 8 Hz, H-3) and 3.57 (1 H, m, H-2); 5.23 (1 H, brs, H-12); ^13^C-NMR (125 MHz; DMSO-*d*_6_): δ_C_ 16.8, 17.1, 23.9, 24.9, 28.9 and 24.5; 64.3 (C-23), 80.5 (C-3), 179.6 (C-28), 122.6 (C-12); 143.9 (C-13).

*Arjunglucoside or 2,3,19,23-tetrahydroxyolean-12-en-28-oïc acid glucopyranoside*. White powder; molecular formula C_36_H_58_O_11_; ESI-MS: [M + Na]^+^ *m*/*z* 689,396; ^1^H-NMR (300 MHz, DMSO-*d*_6_): δ 1.23, 1.08, 0.89, 0.86, 0.84 and 0.63 (each 3 H, s); between 2.90 and 3.80: glucose moiety with anomeric proton at 5,20 (1 H, d, *J* = 6.9 Hz, H-1′); ^13^C-NMR (125 MHz; DMSO-*d*_6_): *δ* 16.9, 24.5, 24.9 and 28.5; glucose moiety: 61.0, 69.9, 72.8, 77.1, 78.2, 94.5; 64.3 (C-23), 67.4 (C-2), 80.4 (C-3), 176.3 (C-28), 122.6 (C-12), 143.7 (C-13).

*2*α*,3*α*,24-trihydroxyolean-11,13(18)-dien-28-oic acid*. Yellowish powder; molecular formula C_30_H_46_O_5_; ESI-MS: [M + Na]^+^ *m*/*z* 509,375 (calc. 509,324) for C_30_H_46_NaO_5_); ^1^H-NMR(400 MHz; pyridin-*d*_5_): δ 1.58; 1.06; 1.05; 1.03; 0.90 and 0.87 (each 3 H, s); 6.62 (1 H, d, *J* = 8.0 Hz, H-11) and 5.81 (1 H, d, *J* = 8.0 Hz, H-12); 4.38 (1 H, ddd, *J* =2.2; 7.6 and 8.9 Hz, H-2); 3.59 (1 H, d, *J* = 7.5 Hz, H-3); 4.43 (1 H, d, *J* = 8.7 Hz, H-24) and 3.75 (1 H, d, *J* = 8,76 Hz, H-24); 2.69 (1 H, d, *J* = 12.4 Hz, H-19) and 2.15 (1 H, d, *J* = 12.4 Hz, H-19); ^13^C-NMR (125 MHz; pyridin-*d*_5_): δ_C_ 16.6, 19.5, 19.8, 23.7, 24.0 and 32.1; 65.1 (C-24), 68.4 (C-2), 85.5 (C-3), 178,6 (C-28);136,1 (C-13); 133,3 (C-18); 126,4 (C-12) et 125,9 (C-11).

Apart from the activity profile described above, MIC values for the other tested fractions and compounds were above 39 µg/ml. The activity level of compounds 3,3′-di-*O*-methylellagic acid 4′-*O*-α-rhamnopyranoside and arjunglucoside was comparable to that of the reference drug fuconazole against *C. albicans*, and compound 3-*O*-methylellagic acid showed to be over 1.5 times more active than the same reference drug against *C. krusei*. Based on the basic skeleton of the tested compounds, it is important to notice that one of the most active derivatives, arjunglucoside and the less active compounds, arjungenin and 2α,3α,24-trihydroxyolean-11,13(18)-dien-28-oic acid are all triterpenoids. Preliminary structure-activity relationship (SAR) study clearly indicated that the glycosylation of the acidic function of arjungenin at C-28 is important for activity improvement. The other active compounds 3,3′-di-*O*-methylellagic acid 4′-*O*-α-rhamnopyranoside and 3-*O*-methylellagic acid are ellagic acid derivatives. Previous studies have reported the antifungal activity of ellagic acid against fungal strains *Trichophyton rubrum*, *T. verrucosum*, *T. mentagrophytes*, *T. violaceum*, *T. schoenleinii*, *Microsporum canis*, *C. glabrata*, *C. albicans* and *C. tropicalis* [41]. Also, the observed antifungal potency of compounds 3,3′-di-*O*-methylellagic acid 4′-*O*-α-rhamnopyranoside and 3-*O*-methylellagic acid, respectively glycosylated and methylated derivatives of ellagic acid, highlights the potency of this class of secondary metabolites [41].

Overall, it was observed that fraction T3 exerted the more potent effect against the tested yeasts, far better than the derived compounds. This is an indication that fractionation has declined the anti-yeast activity, emphasizing the relevance of potential synergistic interactions among the components of fraction T3. Moreover, these results indicate future directions in the progression of this fraction to develop a phytodrug against yeasts infections.

Selected isolated compounds were further tested against G6PD, carbonic anhydrase I, II and GST enzymes. The results achieved are shown in Table 2.

The G6PD enzyme was strongly inhibited by the triterpenoid arjunglucoside with IC_50_ value of 1.84 µM and Ki (the inhibitor constant indicating how potent an inhibitor is; or the concentration required to produce half maximum inhibition) value of 0.19 µM. It has been shown that this key metabolic enzyme which catalyzes the first step of the pentose phosphate pathway is expressed abundantly and is very active in human tumors [21]. In contrast, G6PD-deficient tumor cell lines showed relatively slow growth and enhanced apoptosis [42]. Previous studies also reported G6PD inhibitory properties for few compounds such as steroids and derivatives [43,44], chalcones [29], catechin gallates [45], and some phenolic molecules [46]. In this study, the substituted ellagic acid derived compound 3,3′-di-*O*-methylellagic acid 4′-*O*-α-rhamnopyranoside did not show any effect on the G6PD enzyme activity, although Adem et al. [46] had previously reported that ellagic acid inhibited the enzyme with an IC_50_ value of 0.072 mM. The methoxy group in this derivative may hinder the enzyme–inhibitor interaction. Based on the skeletal features of the tested triterpenoids—arjungenin, arjunglucoside, and 2α,3α,24-trihydroxyolean-11,13(18)-dien-28-oic acid—the presence of the hydroxyl group at C-19 and the glycosylation of the C-28 carboxylic group may be both factors of activity improvement. The G6PD inhibitory potential of a terpenoid is reported here for the first time.

Compound arjunglucoside exhibited very good potency against both CAI and CAII enzymes with respective activity parameters of IC_50_ = 3.28 µM and Ki = 2.72 µM; and IC_50_ = 1.28 µM and Ki = 1.03 µM respectively. The other tested compounds including 3,3’-di-*O*-methylellagic acid 4′-*O*-α-rhamnopyranoside and arjungenin were found to be moderately active against CAI and CAII (3,3′-di-*O*-methylellagic acid 4′-*O*-α-rhamnopyranoside) with IC_50_ and Ki values globally above 44 µM. Previous studies by Sarıkaya et al. [47] have indicated that ellagic acid inhibited CAI and CAII with *K_i_* values of 0.207 and 0.146 mM respectively. In the present study, compound 3,3′-di-*O*-methylellagic acid 4′-*O*-α-rhamnopyranoside, a substituted derivative of ellagic acid has exhibited moderate, however highly improved potency toward CAI (*K_i_* = 44.11 µM) and CAII (55.78 µM) enzymes. On the other hand, this substitution has also considerably decreased the activity as observed against the G6PD enzyme. In addition to the established role of CA inhibitors (CAIs) as diuretics and antiglaucoma drugs, it has recently emerged that they could have potential as novel anti-obesity, anticancer and anti-infective drugs [23]. The high inhibitory potency of the triterpenoid arjunglucoside against CAs indicates that it is a promising compound that might be a candidate for the formulation of drugs against CAIs-related diseases.

The screening of 3,3′-di-*O*-methylellagic acid 4′-*O*-α-rhamnopyranoside, arjungenin, and arjunglucoside against GST enzyme showed inhibitory effects. However, the triterpenoids arjungenin, and arjunglucoside exhibited highly potent inhibitory effects (IC_50_ of 1.57 and 1.84 µM respectively; and *K_i_* of 1.00 and 1.23 µM respectively). Compound 3,3′-di-*O*-methylellagic acid 4′-*O*-α-rhamnopyranoside only exerted a moderate inhibitory effect on the enzyme (IC_50_ = 63.01 µM; *K_i_* = 42.00 µM). These results are of higher significance as GST inhibitors are anti-cancer agents [25,26]. Ellagic acid was recently shown to inhibit GSTs A1-1, A2-2, M1-1, M2-2 and P1-1 with IC_50_ values ranging from 0.04 to 5 µM [48]. Preliminary SAR studies indicate that the substitution of ellagic acid at C-3 and C-4′ gave the derivative 3,3′-di-*O*-methylellagic acid 4′-*O*-α-rhamnopyranoside which showed an IC_50_ value of 63.01 μM, thus therefore considerably decreased the activity. The inhibitory effect of this class of secondary metabolite derivatives is reported here for the first time.

## 5. Concluding Remarks

The results obtained from the investigation of the methanolic extract of *T. mantaly* stem bark have identified a highly potent anti-yeast fraction T3 that showed to be more promising than subsequently isolated compounds. Overall, the five compounds, 3,3′-di-*O*-methylellagic acid 4′-*O*-α-rhamnopyranoside, arjungenin, arjunglucoside, 2α,3α,24-trihydroxyolean-11,13(18)-dien-28-oic acid, and 3-*O*-methyl ellagic acid were found to be 243 to 31,250 times, 15 to 7,812 times, and 975 to 250,000 times less active than the mother fraction (T3) against *C. parapsilosis, C. albicans,* and *C. krusei* respectively. This promising fraction deserves to be further investigated with the ultimate aim of formulating a plant-based drug against yeast infections. Compounds 3,3′-di-*O*-methylellagic acid 4′-*O*-α-rhamnopyranoside and arjunglucoside showed anti-yeast activity close to that of the reference drug fuconazole against *C. albicans.* Moreover, compound 3-*O*-methyl ellagic acid was over 1.5 times more active than fuconazole against *C. krusei*. In addition, two of the islolated compounds, arjungenin and arjunglucoside were found to be very active against enzymes of metabolic significance, namely G6PD (arjunglucoside) and GST (arjungenin and arjunglucoside). Finally, given the anti-yeast potency of these compounds, and also the implication of the tested enzymes in some metabolic dysfunctions of public health significance (cancer, obesity, epilepsy), we envisage further SAR studies to identify potent hit derivatives that should subsequently enter the drug development pipeline.

## Figures and Tables

**Table 1:** Anti-yeast activity of *Terminalia mantaly* extract and isolates.

Extract/Fractions		*C. parapsilosis*	*C. albicans*	*C. krusei*
		MIC * (µg/Ml ± SD)		
MeOH Extract		24.00 ± 0.21	39.00 ± 0.33	39.00 ± 0.30
Fraction T1		1250.00 ± 1.23	2500.00 ± 0.98	2500.00 ± 1.03
Fraction T2		39.00 ± 0.38	>5000	>5000
Fraction T3		0.16 ± 0.02	0.64 ± 0.12	0.02 ± 0.09
Fraction T4		>5000	>5000	>5000
	Fraction of origin			
3,3′-di-*O*-methylellagic acid 4′-*O*-α-rhamnopyranoside	T3	39.00 ± 0.88 (80.4 µM)	9.70 ± 0.72 (20 µM)	>5000 (10,300 µM)
3-*O*-methyl ellagic acid	T3	78.00 ± 0.92 (247.6 µM)	156.00 ± 1.00 (495 µM)	19.50 ± 0.57 (61.9 µM)
Arjungenin	T1, T2, T3	>5000 (9487 µM)	>5000 (9487 µM)	>5000 (9487 µM)
Arjunglucoside	T2, T3	39.00 ± 0.13 (56.60 µM)	9.70 ± 0.36 (14.07 µM)	312.00 ± 1.04 (452 µM)
2α,3α,24-trihydroxyolean-11,13(18)-dien-28-oic acid	T1, T3	>5000 (9823 µM)	>5000 (9823 µM)	>5000 (9823 µM)
Fluconazole **		2.00 ± 0.01 (6.53 µM)	8.00 ± 0.25 (26.14 µM)	32.00 ± 0.42 (10.45 µM)

* Plant extracts were tested using the CLSI M27-A3 protocol. Activity was expressed as minimal inhibitory concentration; ** Reference used as positive control. MIC, minimum inhibitory concentration.

**Table 2:** Inhibitory parameters of isolated compounds against G6PD, CAI, CAII, and GST.

Activity Parameter	G6PD				CAI				CAII				GST			
	1	2	3	4	1	2	3	4	1	2	3	4	1	2	3	4
IC_50_^a^ (µM)	n.a	n.a	n.a	1.84 ± 0.31	53.31 ± 1.09		86.64 ± 0.93	3.28 ± 0.13	69.31 ± 1.13	n.a	n.a	1.03 ± 0.01	63.01 ± 1.15	n.a	1.51 ± 0.78	1.84 ± 0.73
Ki ^b^ (µM)	n.a	n.a	n.a	0.19 ± 0.03	44.11 ± 1.12		71.68 ± 0.96	2.72 ± 0.64	55.78 ± 0.97	n.a	n.a	1.84 ± 0.11	42.00 ± 1.39	n.a	1.00 ± 0.03	0.19 ± 0.77

Enzymes were expressed and purified, and subsequently assessed for in vitro susceptibility to inhibitors. ^a^ Serially diluted triplicate concentrations of compounds were tested and activity expressed as 50% inhibitory concentration; ^b^ Inhibitory constant which is reflective of the binding affinity; the smaller the Ki, the greater the binding affinity and the smaller amount of medication needed in order to inhibit the activity of that enzyme. n.a = non active. 1: 3,3′-di-*O*-methylellagic acid 4′-*O*-α-rhamnopyranoside; 2: 3-*O*-methylellagic acid; 3: arjungenin; 4: arjunglucoside. G6PD, glucose-6-phosphate dehydrogenase; CAI, human erythrocyte carbonic anhydrase I; CAII, human erythrocyte carbonic anhydrase II; GST, glutathione *S*-transferase.
